# Functional training added to intradialytic cycling lowers low-density lipoprotein cholesterol and improves dialysis adequacy: a randomized controlled trial

**DOI:** 10.1186/s12882-020-02021-2

**Published:** 2020-08-18

**Authors:** Špela Bogataj, Jernej Pajek, Jadranka Buturović Ponikvar, Maja Pajek

**Affiliations:** 1 Department of Nephrology, University Medical Centre, 1000 Ljubljana, Slovenia; 2 University of Ljubljana, Faculty of Sport, 1000 Ljubljana, Slovenia; 3 University of Ljubljana, Faculty of Medicine, 1000 Ljubljana, Slovenia

**Keywords:** Exercise, Hemodialysis, CKD, Intervention, Health promotion

## Abstract

**Background:**

Exercise has various positive effects on hemodialysis patients. However, there is no clear evidence which type of exercise yields better results. This study aimed to determine the effects of guided functional training added to the intradialytic cycling on dialysis adequacy and biochemical parameters in hemodialysis patients. Additionally, we aimed to investigate if patients could transfer functional exercise to an unsupervised home environment and retain gained improvements.

**Methods:**

Randomization was done to a functional training intervention group (INT) (*n* = 20) or intradialytic cycling control group (CON) (n = 20). The INT attended a pre-dialysis functional training in the first 8 weeks. In the second 8 weeks, they performed functional exercises at unsupervised home environment on non-dialysis days. During the whole study, both groups participated in the intradialytic cycling program.

**Results:**

Both groups demonstrated a significant increase in dialysis adequacy (Kt/V) in the eight (0.15, 95% CI 0.06 to 0.24; *p* = 0.003 for INT and 0.21, 95% CI 0.11 to 0.3; *p* < 0.001 for CON) and the 16th study week (0.13, 95% CI 0.03 to 0.24; *p* = 0.017 for INT and 0.13, 95% CI 0.03 to 0.22; *p* = 0.013 for CON) compared to their baseline values with no significant between-group differences. At week eight, the total cholesterol was significantly lowered in the INT (− 0.34 mmol/L, 95% CI − 0.6 to − 0.07; *p* = 0.016) and remained lower at week 16 (− 0.32 mmol/L, 95% CI − 0.64 to − 0.01; *p* = 0.049) with no significant changes in the CON. Low-density lipoprotein levels in the INT were significantly reduced after 8 weeks (− 0.35 mmol/L, 95% CI − 0.64 to − 0.06; *p* = 0.022) and remained reduced after 16 weeks (− 0.28 mmol/L, 95% CI − 0.52 to − 0.03; *p* = 0.030). There were no significant differences found for albumin, high-density lipoprotein cholesterol, triglycerides, C-reactive protein, and hemoglobin in both groups.

**Conclusions:**

We demonstrated that functional training added to intradialytic cycling improved lipid profile and dialysis adequacy. Additionally, the effects of the unsupervised, home-based program were preserved during the second study phase. This study supports the assumption that combined training is more effective compared to solely intradialytic exercise.

**Trial registration:**

ClinicalTrials.Gov, NCT03334123. Registered 07 November 2017.

## Background

Chronic kidney disease (CKD) is an important and common cause of death with the increasing number of patients around the world [1]. CKD is related to obesity, type 2 diabetes, arterial hypertension and confers increased cardiovascular disease risk [2]. The majority of patients with moderate CKD die before they reach end-stage renal disease (ESRD) [3]. However, increased physical activity could have a survival benefit for CKD patients and subsequently reduce their death rate [4, 5].

Multiple studies [6–23] have investigated physical exercise interventions as a treatment for CKD. Although the adequacy of exercise modalities in these studies is inconsistent [24], Johansen & Painter [25] have noted that physical exercise appears to be safe in the CKD population, with no single study reporting the worsening of kidney function. Moreover, several systematic reviews and meta-analyses published recently delivered the consensus that regular exercise is beneficial for patients in CKD stages 1–4 and in end-stage renal disease (CKD stage 5), with the majority of studies conducted in CKD stage 5 patients treated with hemodialysis (HD) [15, 26–31].

Intradialytic cycling performed during HD has been widely accepted as a beneficial exercise intervention due to its feasibility and improvements in physical performance [6, 7, 32]. However, integrating intradialytic cycling with an aerobic and resistance exercise program to increase the volume and quality of exercise (e.g., functional training) could improve hematological indexes, health-related quality of life, and reduce inflammatory cytokines and depression [17]. Studies have also provided evidence of intradialytic exercise programs enabling improvement in urea clearance (Kt/V) [16], reduced need for antihypertensive medications [18], an increase in hemoglobin concentration, and improvement in lipid metabolism [19]. It is well documented that people with CKD have a higher risk of developing cardiovascular decease [33]. Therefore, normalisation or at least aiming to reduce the elevated levels of low-density lipoprotein (LDL) should be of great importance for CKD patients. However, the impact of exercise interventions for lowering LDL cholesterol is not sufficiently defined [21–23]. Goldberg et al. [22] and more recently Cheema et al. [21] showed reduced levels of LDL cholesterol in HD patients, while De Moura et al. [23] found increased levels of LDL and triglycerides following aerobic exercise during HD. Therefore, we need additional evidence about effectiveness of exercise interventions in dialysis patients targeting LDL cholesterol levels to prevent the development of cardiovascular decease.

It is stated that an HD treatment is »adequate« when patients have a good nutritional condition, are cleared from uremia symptoms, have normal blood pressure, satisfactory red blood cell production, and are prevented from neuropathy development [34]. The most frequently used small solute HD adequacy indicator is the urea Kt/V. It stands for the product of urea clearance and time of HD procedure per unit of urea distribution volume [35]. A higher Kt/V represents better small solute removal, which is linked to lower organ toxicity. Exercise training could improve Kt/V by reducing the post-dialysis rebound in concentration of uremic solutes [36]. The reduced urea rebound and improved Kt/V is probably mediated by an increase of intradialysis muscle and systemic blood flow [36]. On the contrary, intradialysis exercise on a cycle ergometer distributed in 15-min intervals during each of the first 3 h of HD session resulted in an insufficient magnitude of increase in overall Kt/V [14]. Accordingly, we need additional studies of various exercise types and delivery methods to optimize the utilization of intradialysis exercise.

The majority of the mentioned studies were limited to three types of exercise: aerobic exercise, resistance exercise, and combined (aerobic and resistance) exercise. Since most of the evidence exists on the effectiveness of intradialytic aerobic training, it would be beneficial to investigate the effects of different delivery types of exercise, such as home-based, intradialytic, and non-intradialytic exercise. Moreover, physical exercise appears to have inconsistent benefits in HD patients, presumably due to variable intensity and exercise volumes. Consequently, innovative approaches with individualized exercise prescription and lifestyle interventions are needed. Due to a lack of staff who are qualified in prescription and follow-thorough of physical exercise, we should also seek for effective strategies to prepare patients for independent physical exercise in their home environment. The best results may be obtained with the professional dedication of a multidisciplinary team to ensure sustainable physical exercise programs as part of a routine care for HD patients. However, the identification of an optimized, individualized training program is yet to be made.

The present study aimed to determine the effects of functional training and exercise counseling added to the intradialytic cycling program on dialysis adequacy and biochemical parameters in HD patients. Additionally, we aimed to investigate the sustainability and effects of functional exercise transfer to an unsupervised home environment on non-dialysis days and the level of retention of improvements from the first (supervised exercise) study phase.

## Methods

**Fig. 1:**
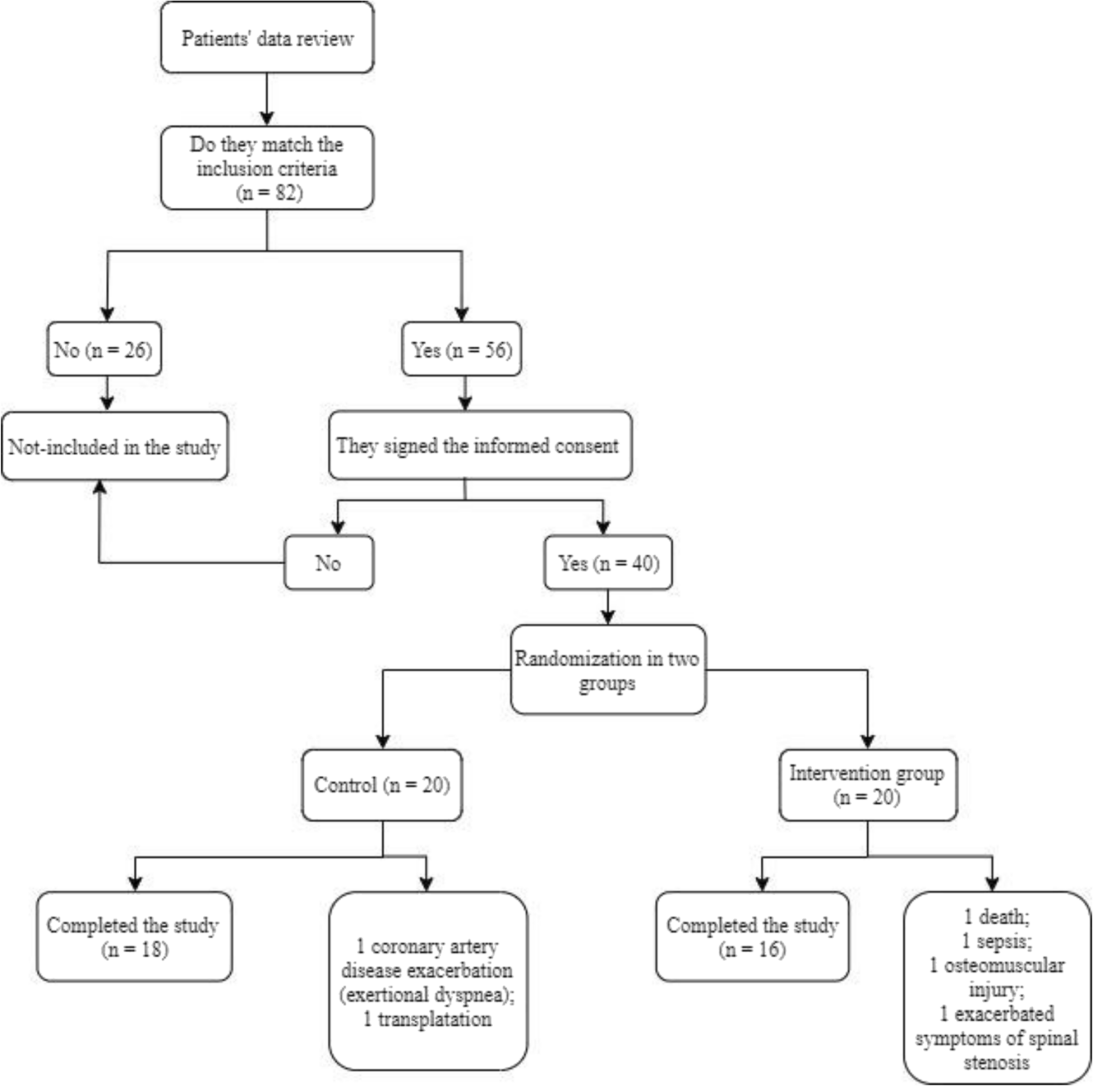
Study flow diagram

The present prospective, randomized, controlled trial was conducted on 40 voluntary HD patients at HD units of the University Medical Centre in Ljubljana, Slovenia. Patients were randomized from November 2017 to February 2019 into the intervention and control group. Exercise interventions and protocols were described in detail previously [37]. The intervention group engaged in guided functional exercise training before each HD session and additional intradialytic cycling exercise in the first 8 weeks. In the second 8 weeks, pre-dialysis functional training was dismissed, and patients were instructed, motivated, and encouraged to perform functional exercise routines at home on non-dialysis days. The exercise program of intradialytic cycling was continued. During both study periods, the control group performed intradialytic cycling only. The flow of the subjects is presented in Fig. 1. The trial compared the effects of the mentioned exercise training strategies on biochemical parameters and dialysis adequacy.

### Study criteria and ethical considerations

The inclusion criteria limited the selection to patients on renal replacement therapy with HD longer than 3 months, age 18–90 years, and a stable medical condition. Exclusion criteria were an infectious or chronic malignant disease, uncontrolled arterial hypertension, angina pectoris grade 2–4 (Canadian Cardiovascular Society), heart failure grade 3 or 4 (New York Heart Association), the presence of a mental disability, limb amputation (two or more fingers on the lower limb and/or on the upper limb). Withdrawal criteria were acute intercurrent illness or trauma that lasted longer than 14 days, the occurrence of malignant disease during the study period, and voluntary withdrawal from the study. National Medical Ethics Committee approval was attained from the Ministry of Health, Republic of Slovenia (approval document number 0120–97/2017–3 KME 68/03/17). All patients signed written informed consent and were informed about the aim, confidentiality, and procedures of the study. The study complies with the Declaration of Helsinki (1964). The study was registered at ClinicalTrials. Gov (Clinicaltrials.gov identifier: NCT03334123).

### Study design and outcomes

The primary study end-point was urea Kt/V per single dialysis episode. Secondary study outcomes were metabolic and inflammatory biochemical parameters. The total length of the intervention was 16 weeks, divided into two phases. Biochemical parameters were assessed at baseline, after 8 weeks, and after 16 weeks at the beginning of each week (Table 2). We assessed albumin, total cholesterol, high-density lipoprotein cholesterol (HDL), low-density lipoprotein cholesterol (LDL), triglycerides (TG), C-reactive protein (CRP), and hemoglobin. Levels of each parameter were determined with routine laboratory methods in a local laboratory. Dialysis adequacy (Kt/V) was determined as the average of measurements at first and at the second dialysis session of the week on the HD machine Fresenius Medical Care (dialysis monitor type 5008®). An online clearance monitoring system was used, which measures the Kt/V value that corresponds to a single pool Kt/V value and is measured by sodium conductivity variation [38]. “V” term (distribution volume for urea) was determined using bioimpedance analysis (Body Composition Monitor®, Fresenius Medical Care, Bad Homburg, Germany). Kt/V was assessed in the first week, in the 8th week and the 16th week of the intervention.

### Interventions description

**Fig. 2:**
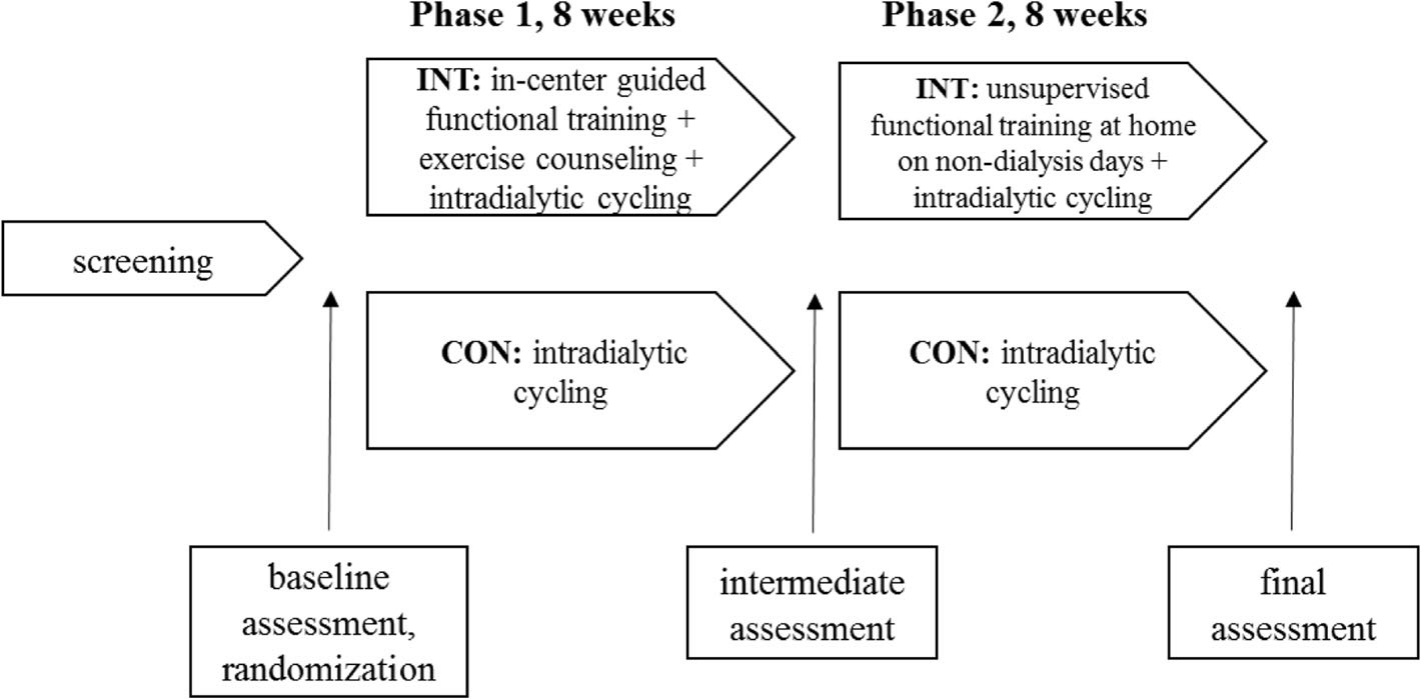
Study overview. Abbreviations: INT, intervention group; CON, control group

After the baseline assessment, the patients were randomized following simple randomization procedures (computerized random numbers) and allocated into the intervention group (INT) or the control group (CON). We allocated patients after baseline assessment and following a list assessed only by the main researcher (and not treating physicians) to avoid selection bias. An overview of the study procedure is shown in Fig. 2. In the first study phase (first 8 weeks), the intervention group attended a guided functional training three times a week before the HD procedure. Besides training, they also received exercise counseling to acquire the functional exercise skills accurately and transfer them to their home environment in the second study phase (second 8 weeks). During the first 2 h of dialysis, they additionally performed a cycling exercise on the customized bike (Model B’fit Mini, Lemco®, Denmark). The control group performed the same cycling program as the intervention group without pre-dialysis functional training and counseling.

Functional training was performed for approximately 30 min before each dialysis session. The intensity was set to 7th to 8th grade on a 10-grade Borg scale. The intradialytic exercise started with 15 min of cycling and was gradually increased in duration (up to 60 min) and intensity to maintain the intensity of 4th to 5th grade on a 10-grade Borg scale. Both types of exercise, including exercise counseling, were guided and prescribed by a kinesiologist. Functional training was tailored to the individual’s capacity and included exercises with a full range of motion with additional weights. Light cardiovascular, coordination and balance exercises were performed in the warm-up. In the main part of the functional training, patients performed different varieties of lunges, squats, pulls, push-ups, lifts, and pushes. The cool-down part included light cardiovascular exercises combined with stretching. Exercise counseling was given at the time of functional training. The patients received instructions on how to correctly perform an exercise, how to modify an exercise, and how to adjust the resistance/load.

In the second phase, the intervention group transferred the pre-dialysis functional training to their home environment. The kinesiologists prepared for each patient written and illustrated exercise program with whom they could track their home exercise and mark the exercises that they performed. The prescribed exercises are listed in Additional file 1. On dialysis days, we assessed compliance on the basis of their self-report and discussed the issues of home functional exercise, giving feedback, counseling, and motivation. At every dialysis session of the second study phase, they reported the details about the exercise performed during the previous inter-dialysis period. We focused on motivating the patients to stay engaged in the exercise process by discussing the barriers to exercise, setting goals, monitoring safety, and identifying and solving intercurrent problems. Intradialytic cycling program remained the same for both groups during the whole second phase. The kinesiologist and nursing staff monitored patients for complications or adverse effects (e.g., dyspnea, headache, fatigue, chest, muscle, or joint pain) throughout the exercise sessions. Participant blood pressure and heart rate were measured at the beginning and the end of functional training and in the beginning, after 15 min, and immediately after the intradialytic cycling session.

### Statistical analysis

Descriptive statistics (mean ± SD) were calculated for sex, age, weight, height, dialysis vintage, weekly dialysis duration, systolic, and diastolic blood pressure. The independent t-test was used to compare the group’s baseline demographic and clinical characteristics. Analysis of covariance (ANCOVA) was used to test for differences between the groups with the baseline value as a covariate. A paired t-test was used to analyze within-group changes over time. We included in analysis all patients available for biochemical parameters assessment, physical performance testing, and Kt/V measurement except for injured, hospitalized, transplanted or deceased patients where these measurements were not feasible (for details see Fig. 1). All tests were 2-sided, performed using the SPSS program (version 22; SPSS Inc., Chicago, IL, USA), and assessed at the *p* < 0.05 level of significance.

## Results

### Patients’ characteristics and exercise adherence

Table 1 outlines the patients’ baseline demographic and clinical characteristics. There were no statistically significant differences between the groups.

**Table 1:** Comparison of demographic and clinical characteristics between the intervention and control group

Variable	Intervention group (*n* = 20)	Control group (*n* = 20)	***P***-value
Age (years)	65.2 ± 12.1	61.9 ± 13.0	0.43
Male sex (n)	12 (60%)	10 (50%)	0.53
Weight (kg)	72.6 ± 16.1	71.7 ± 15.9	0.42
Height (cm)	168.4 ± 9.6	167.5 ± 10.2	0.42
Dialysis vintage (years)	7.4 ± 8.1	7.5 ± 7.3	0.43
Weekly dialysis duration (h)	12.5 ± 2.7	13.3 ± 1.9	0.43
Systolic blood pressure (mm Hg)	141 ± 16.1	144 ± 14.93	0.50
Diastolic blood pressure (mm Hg)	78 ± 10.2	84 ± 9.38	0.81

Note: Values are expressed as mean ± SD or number of subjects (percent). Blood pressure was defined as the mean of the last three pre-dialysis blood pressure values. Abbreviations: *n* number of subjects

Adherence to training programs was defined as the total number of completed exercise sessions in contrast to the total number of sessions offered. In phase 1, adherence of the intervention group for functional training and cycling sessions were 87% ± 12 and 90% ± 12%, respectively. In the 8th week, after a gradual increase in duration, the mean intra-dialytic cycling routine duration was 30.5 ± 8.3 min for the intervention group and 31.8 ± 7.8 min for the control group (*p* > 0.05). In the 16th study week, the intervention group cycled for 46.6 ± 17.0 min and control group for 44.4 ± 12.8 min (*p* > 0.05). In phase 2, the intervention group completed 73% ± 21% of advised at home functional exercise sessions and 82% ± 19% of in-center cycling sessions. Control group adherence in phase 2 was 82% ± 13% (cycling sessions). There were no significant between-group differences in intradialytic cycling adherence during either study phases. However, there was a significantly (*p* = 0.034) lower adherence to home-based functional exercise in contrast to in-center pre-dialysis functional training in the intervention group.

### Changes in dialysis adequacy

**Fig. 3:**
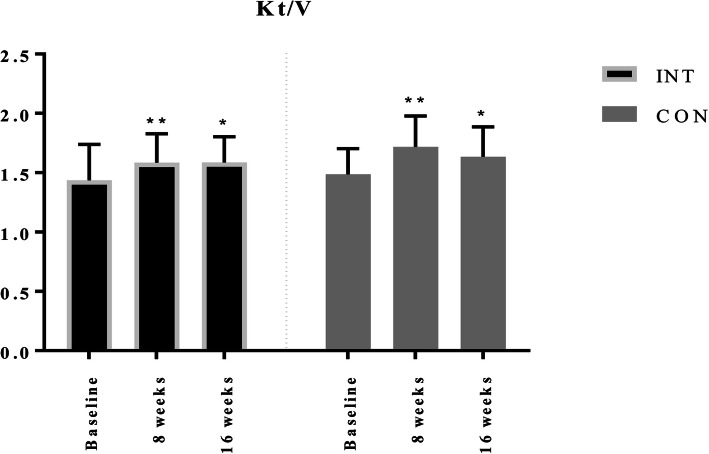
Within-group changes in Kt/V value. Note: *, *p* < 0.05 indicates significant within-group difference compared to the baseline value. **, *p* < 0.01 indicates significant within-group difference compared to the baseline value. Abbreviations: INT, intervention group; CON, control group; Kt/V, dialysis adequacy

Within-group changes in dialysis adequacy (Kt/V) are presented in Fig. 3. There were no statistically significant differences in blood flow, filter size, and duration of the HD session between groups and between study phases (Table 2). Both groups demonstrated a significant increase in urea Kt/V at the 8th and the 16th week of the study compared to their baseline values. Intervention group improved Kt/V from 1.45 ± 0.25 to 1.60 ± 0.24 at week eight (0.15 ± 0.17, 95% CI 0.06 to 0.24; *p* = 0.003) and to 1.59 ± 0.22 at week 16 (0.13 ± 0.2, 95% CI 0.03 to 0.24; *p* = 0.017). The baseline Kt/V value in the control group was 1.51 ± 0.21 and raised to 1.72 ± 0.26 at week eight (0.21 ± 0.18, 95% CI 0.11 to 0.30; *p* < 0.001) and to 1.64 ± 0.25 at week 16 (0.13 ± 0.18, 95% CI 0.03 to 0.22; *p* = 0.013). As both groups significantly improved, there was no significant between-group difference in the 8th week (baseline adjusted *p* = 0.267) and also in the 16th week of the study (*p* = 0.874).

**Table 2:** Dialysis adequacy parameters

Group	Blood flow (ml/min)			Filter size (m^**2**^)			Duration of the HD session (min)		
	Baseline	8th week	16th week	Baseline	8th week	16th week	Baseline	8th week	16th week
**INT**	302.8 ± 8.7	304.4 ± 18.3	301.7 ± 15.6	2.0 ± 0.4	2.0 ± 0.3	2.1 ± 0.3	267.8 ± 31.0	277.2 ± 30.6	278.9 ± 27.7
**CON**	287.3 ± 30.4	293.5 ± 29.4	289.6 ± 29.3	1.9 ± 0.3	1.9 ± 0.3	1.9 ± 0.3	277.7 ± 21.7	277.3 ± 29.0	267.5 ± 21.2

Note: Values are expressed as mean ± SD. There were no statistically significant differences between groups nor between study phases. Abbreviations: *INT* intervention group, *CON* control group

### Within-group changes in biochemical parameters

No significant differences were found for albumin, HDL, triglycerides, CRP, and hemoglobin for both groups at the end of either study phases (Table 3). At week eight, the total cholesterol was significantly lowered in the intervention group (− 0.34 ± 0.51 mmol/L, 95% CI − 0.6 to − 0.07; *p* = 0.016) and remained lowered at week 16 (− 0.32 ± 0.61 mmol/L, 95% CI − 0.64 to − 0.01; *p* = 0.049) with no significant differences in the control group. LDL did not significantly change in the control group; however, intervention group showed a significant decrease in LDL in the 8th week (− 0.35 ± 0.56 mmol/L, 95% CI − 0.64 to − 0.06; *p* = 0.022) and the 16th week (− 0.28 ± 0.46 mmol/L, 95% CI − 0.52 to − 0.03; *p* = 0.030) of the study.

**Table 3:** Within-group comparison of biochemical parameters

Variable	Intervention group			Control group		
	Baseline	8th week	16th week	Baseline	8th week	16th week
Albumin (g/L)	39.9 ± 3	40.2 ± 3.3	40.0 ± 2.5	39.5 ± 2.7	40.3 ± 3.3	39.9 ± 3.2
Cholesterol (mmol/L)	4.53 ± 0.91	4.18 ± 0.95*	4.21 ± 0.86*	4.23 ± 1.31	4.06 ± 1.03	4.24 ± 1.31
LDL (mmol/L)	2.39 ± 0.77	1.99 ± 0.81**	2.12 ± 0.69*	2.13 ± 1.12	1.96 ± 0.79	2.28 ± 0.92
HDL (mmol/L)	1.28 ± 0.46	1.33 ± 0.62	1.24 ± 0.38	1.23 ± 0.48	1.31 ± 0.53	1.26 ± 0.52
TG (mmol/L)	1.85 ± 1.06	1.86 ± 1.28	2.11 ± 1.49	2.14 ± 3.05	1.93 ± 2.01	1.66 ± 1.05
CRP (mg/L)	4.37 ± 4.27	3.73 ± 2.15	4.50 ± 4.53	4.72 ± 4.21	4.50 ± 3.92	6.21 ± 5.87
Hemoglobin (g/L)	118.63 ± 7.60	117.94 ± 8.86	117.94 ± 8.86	122.22 ± 11.95	118.56 ± 17.69	118.56 ± 17.69

Note: Values are expressed as mean ± standard deviation. *, *p* < 0.05 indicates significant within-group difference compared to the baseline value. **, *p* < 0.01 indicates significant within-group difference compared to the baseline value. Abbreviations: *LDL* low-density lipoprotein cholesterol, *HDL* high-density lipoprotein cholesterol, *TG* triglycerides, *CRP* C-reactive protein

### Between-group changes in biochemical parameters

Baseline adjusted ANCOVA analyses (Table 4) revealed a significant between-group mean difference for LDL in the 16th week of the study. The difference was − 0.35 ± 0.16 mmol/L (CI 95% -0.68 to − 0.03) in favor of the intervention group.

**Table 4:** The between group difference in biochemical end-points

	8th week		16th week	
Variable	Difference (95% CI)	p-value (INT-CON)	Difference (95% CI)	p-value (INT-CON)
Albumin (g/L)	−0.48 ± 0.73 (− 1.97 to 1.02)	0.520	−0.18 ± 0.66 (− 1.51 to 1.16)	0.79
Cholesterol (mmol/L)	− 0.11 ± 0.17 (− 0.46 to 0.26)	0.574	−0.23 ± 0.24 (− 0.71 to 0.25)	0.34
LDL (mmol/L)	−0.16 ± 0.16 (− 0.48 to 0.17)	0.339	−0.35 ± 0.16 (− 0.68 to − 0.03)	0.03*
HDL (mmol/L)	−0.03 ± 0.06 (− 0.15 to 0.09)	0.611	−0.05 ± 0.08 (− 0.22 to 0.11)	0.51
TG (mmol/L)	0.12 ± 0.19 (− 0.28 to 0.52)	0.537	0.56 ± 0.33 (− 0.12 to 1.24)	0.11
CRP (mg/L)	0.53 ± 0.71 (− 0.93 to 1.98)	0.464	− 1.18 ± 2.03 (− 5.35 to 2.99)	0.57
Hemoglobin (g/L)	0.84 ± 4.84 (− 9.03 to 10.72)	0.863	−0.84 ± 4.12 (− 9.24 to 7.56)	0.84

Note: Values are expressed as adjusted mean difference ± standard deviation using ANCOVA – analysis of covariance with adjustment for baseline values. All significant between-group differences with ANCOVA adjusted test were also significant with unadjusted between-group ANOVA test (*p* < 0.05). Abbreviations: *INT* intervention group, *CON* control group, *LDL* low-density lipoprotein cholesterol, *HDL* high-density lipoprotein cholesterol, *TG* triglycerides, *CRP* C-reactive protein, *Kt/V* dialysis adequacy, *ANCOVA* analysis of covariance, *CI* confidence interval; *, *p* < 0.05

## Discussion

The main findings in this study were that 8 weeks of functional training added to intradialytic exercise lowered total cholesterol and LDL in the intervention group while a significant increase in urea Kt/V was observed in both groups following 8 and 16 weeks of training, with no difference between both exercise programs. Within-group changes in cholesterol and urea Kt/V were sustained after the transfer of training to an unsupervised home environment.

This study demonstrates that both studied exercise programs improve small solute dialysis adequacy. At the end of the 8th week, patients presented a significant increase in Kt/V in both groups. This increase in the Kt/V remained significant after 16 weeks in the control group as well as in the intervention group. Exercising during HD procedure leads to an increase in muscle blood flow, with a consequential increase in diffusion area, serum urea clearance and improvement in dialysis adequacy [14]. We speculate that the addition of a functional training did not differ in Kt/V improvements from aerobic cycling exercise alone, because the functional exercise was not performed during the dialysis and did not alter these mechanisms. The aforementioned increase in muscle blood flow during dialysis exercise opens the capillary surface area, consequently increasing the diffusive flux of urea to the vascular compartment [14]. This process increases serum urea clearance and improves dialysis adequacy. Our results agree with the findings of Parsons et al. [14] and Mohseni et al., [39] and demonstrate the benefit in dialytic small solute clearance also with the addition of pre-dialysis training and further with unsupervised training on non-dialysis days as long as the exercise program contains intradialytic exercise. A recent meta-analysis [20] showed improvements in dialysis adequacy following exercise training in HD patients. A single intradialytic cycling session of 60 min at submaximal exertion improved Kt/V by 14%, which is comparable to the effect of 20 min prolongation of HD treatment time [36].

Patients in the last stage of chronic kidney disease usually have either normal or increased LDL and decreased levels of HDL [40]. There is clear evidence that lower LDL in population with normal or slightly altered kidney function is beneficial in preventing major cardiovascular risks and mortality [41]. However, in dialysis patients, LDL cholesterol shows a negative association with cardiovascular outcomes at below-average LDL cholesterol levels and a flat or weakly positive association with mortality at higher LDL cholesterol levels [42]. It is known that aerobic exercise can reduce LDL and increase HDL levels in patients on HD [21, 22]. De Moura et al. [23], on the contrary, in a study that included 12 weeks of supervised aerobic exercise training during HD showed an increase in triglycerides and LDL. In the present study, there was a significant decrease in total cholesterol and LDL concentrations in the intervention group, with a significant difference in LDL cholesterol between groups after 16 weeks. While KDOQI guidelines do not advocate de-novo statin therapy for lipid-lowering in dialysis patients [43], this does not extend automatically to lipid-lowering with exercise interventions, which provide other important benefits beyond lipid control. It is well documented that the addition of resistance training to aerobic exercise can improve the lipid profile [44]. This was confirmed in a recent study, which showed that interdialytic endurance-resistance training reduced triglycerides and LDL levels and increased HDL [45]. Our findings suggest that with increased volume of exercise in the INT group, there may be a significant effect on total cholesterol and especially on LDL, opposite to a more limited exercise volume in the CON group. Additionally, one study showed that high-load intradialytic resistance training improved lean leg mass in HD patients [46]. Having in mind that cholesterol is associated with skeletal muscles’ response [47], it was expected that LDL levels would reduce in the current study. The 0.35 mmol/l between-group difference is much smaller than expected with a statin therapy where LDL lowering in the range of 0.85–1.27 mmol/l difference was found in major trials [42].

This study has some limitations that should be mentioned. First, the sample size was relatively small, which influenced controlling for several factors that may have impacted the results. Second, the intervention was not long enough to tentatively improve some markers of inflammation. Furthermore, the functional training group had individual attention and additional motivation, which could influence their improvements. There was a lack of an inactive control group to compare between-group differences with functional training group and group who only performed intradialytic cycling. Moreover, we did not measure nutritional intakes in this study, so we were unable to analyse the association of lipid profile or physical performance improvements with differences in each individual’s nutritional composition. Observed adverse events during the study were mostly fatigue, joint and low back pain, and isolated hypotension. No major cardiac events and instability in vital signs were observed. Future studies should include larger sample size and longer interventions to be able to see benefits associated with functional training.

## Conclusions

In summary, we demonstrated that 8 weeks of functional training added to intradialytic cycling improved dialysis adequacy and lipid profile. Additionally, the effects of the subsequent unsupervised, home-based program in the functional training group were preserved during the second phase when the transfer of mastered exercise routines to the home environment was done. These data give evidence on the novel exercise prescription strategy by which combined pre-dialysis functional exercise training and intradialytic exercise may increase small solute clearance and, at the same time, reduce cardiovascular risks by improving lipid profile in patients with CKD.

## Supplementary information

**Additional file 1 Table S1.** Exercises prescribed in the functional training program.

## Data Availability

Data generated and analyzed during this study are included in this article. Additional data are available from the corresponding author on request.

## Abbreviations

INT: Intervention group
CON: Control group
HDL: High-density lipoprotein cholesterol
LDL: Low-density lipoprotein cholesterol
TG: Triglycerides
CRP: C-reactive protein
CKD: Chronic kidney disease
HD: Hemodialysis
Kt/V: Dialysis adequacy
SD: Standard deviation
ANCOVA: Analysis of covariance
n: Number of subjects
CI: Confidence Interval
