# Supplementary material for: Functional training added to intradialytic cycling lowers low-density lipoprotein cholesterol and improves dialysis adequacy: a randomized controlled trial
Source: BMC Nephrol. 2020 Aug 18;21:352. doi: 10.1186/s12882-020-02021-2 (PMC7436960; doi:10.1186/s12882-020-02021-2)
Supplement: Supplementary file 1 — Additional file 1 Table S1. Exercises prescribed in the functional training program. [file 12882_2020_2021_MOESM1_ESM.docx]

**Additional file 1**

Table S1. Exercises prescribed in the functional training program

| **Warm-up** | **Main part** | **Cool-down** |
| --- | --- | --- |
| Light cardiovascular exercises and exercises for coordination and balance;  - walking in place  - walking heel to toe  - walking on toes  - walking backward  - knee lifts  - heel raises  - shoulder circles  - arm circles  - arm swings  - trunk twists  - leg swings forward  - ankle circles | - Squat: chair squat, wall squat with an exercise ball, squat, squat with dumbells  - Lunges: stationary lunge, lunge with a chair, walking lunge, reverse lunge, rear/front foot elevated lunge, lunge with dumbbells  - Push-ups: wall push-ups, incline push-ups, knee push-ups, regular push-ups  - Pull exercises: horizontal and vertical pull with a resistance band, trx, dumbbells  - Leg raises: back, and side raises (adjusting load with resistance bands and ankle weights)  - Pallof press, modified side plank, sit-ups, glute bridges, seated rotations, bird dog, chest squeeze with a medicine ball, dead bug | - palm press up toward the ceiling  - calf stretches  - abdominal stretch  - shoulder stretch  - hip flexor stretch  - lower back stretch  - quadriceps stretch  - biceps stretch  - seated forward bend  - knee-to-chest pose |
